# Locking Two Rigid-body Bundles in an Outward-Facing Conformation: The Ion-coupling Mechanism in a LeuT-fold Transporter

**DOI:** 10.1038/s41598-019-55722-6

**Published:** 2019-12-20

**Authors:** Jing Li, Zhiyu Zhao, Emad Tajkhorshid

**Affiliations:** 1 NIH Center for Macromolecular Modeling and Bioinformatics, University of Illinois at Urbana-Champaign, Urbana, IL 61801 United States; 2 Beckman Institute for Advanced Science and Technology, University of Illinois at Urbana-Champaign, Urbana, IL 61801 United States; 3 Department of Biochemistry, University of Illinois at Urbana-Champaign, Urbana, IL 61801 United States; 4 Center for Biophysics and Quantitative Biology, University of Illinois at Urbana-Champaign, Urbana, IL 61801 United States; 5 Present Address: Department of Biochemistry and Molecular Biology, University of Chicago, Chicago, IL 60637 United States

**Keywords:** Computational biophysics, Computational models

## Abstract

Secondary active transporters use electrochemical gradient of ions to fuel the “uphill” translocation of the substrate following the alternating-access model. The coupling of ions to conformational dynamics of the protein remains one of the least characterized aspects of the transporter function. We employ extended molecular dynamics (MD) simulations to examine the Na^+^-binding effects on the structure and dynamics of a LeuT-fold, Na^+^-coupled secondary transporter (Mhp1) in its major conformational states, i.e., the outward-facing (OF) and inward-facing (IF) states, as well as on the OF ↔ IF state transition. Microsecond-long, unbiased MD simulations illustrate that Na^+^ stabilizes an OF conformation favorable for substrate association, by binding to a highly conserved site at the interface between the two helical bundles and restraining their relative position and motion. Furthermore, a special-protocol biased simulation for state transition suggests that Na^+^ binding hinders the OF ↔ IF transition. These synergistic Na^+^-binding effects allosterically couple the ion and substrate binding sites and modify the kinetics of state transition, collectively increasing the lifetime of an OF conformation with high substrate affinity, thereby facilitating substrate recruitment from a low-concentration environment. Based on the similarity between our findings for Mhp1 and experimental reports on LeuT, we propose that this model may represent a general Na^+^-coupling mechanism among LeuT-fold transporters.

## Introduction

Active transporters are membrane proteins evolved to utilize various forms of cellular energy to efficiently transport selective substrates across the membrane against their electrochemical gradient. Distinguished from ATP-powered, primary active transporters, secondary active transporters harness transmembrane electrochemical gradient of one solute to drive uphill translocation of another^1–5^. Many of these transporters, for which various ions (most prominently Na^+^ or H^+^) serve as the energy-providing solutes, are therefore termed ion-coupled secondary active transporters^6^. In order to furnish their function, all ion-coupled transporters operate following the alternating-access model, in which during the transport cycle the transporter protein switches between two major conformational states, an inward-facing (IF) and an outward-facing (OF) one, to alternate the substrate access between the two sides of the membrane^7–9^. The alternating-access model has received substantial support from a large number of structural studies of membrane transporters^5,6,10–14^.

**Figure 1:**
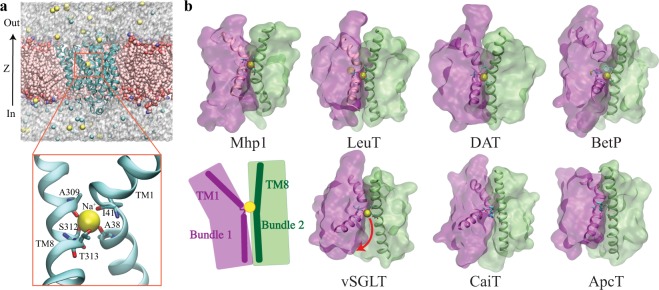
A conserved ion-binding site (Na2 site) in LeuT-fold transporters. (**a**) Overview of the structure of Mhp1 and the simulation system. (top) The simulation system. Mhp1 is rendered in cartoon, with the bound Na^+^ drawn in VDW. The POPE lipids and the solute ions are also drawn in VDW, and the water molecules are represented as semi-transparent surfaces. Lipids molecules overlapping with the protein are hidden for clarity. (bottom) A close view of the highly conserved Na^+^-binding site (Na2). Residues surrounding the Na^+^-binding site along with helices TM1 and TM8 are shown and labeled. (**b**) The Na2 site is a conserved motif shared by Na^+^-dependent transporters, namely, Mhp1, LeuT, DAT, BetP and vSGLT, and is replaced by a basic residue with a similar role in two Na^+^-independent transporters: CaiT and ApcT. The top panel shows four transporters in their OF states, with a well-coordinated Na2 site, while the bottom panel shows three transporters in either IF or occluded state with a more open Na2 site. TM1 and TM8 are represented in cartoon form, with the bound Na^+^ drawn in VDW. The two helical bundles are shown in transparent surface representation. The binding residues in Na2 site are shown in sticks. Bottom panel also includes a schematic summarizing the common architecture among the proteins shown, namely, a conserved site housing a positive charge and formed at the interface of the two helical bundles by TM1 and TM8 helices.

Based on a number of solved crystal structures^15–33^, several secondary active transporter families were surprisingly identified to bear remarkable architectural resemblance—two inverted-topology repeats of five-transmembrane-helices (TMs) bundles that are oppositely oriented with respect to the membrane, with the first helix of each repeat always partially unwound^34^. This architecture is now considered to represent a superfamily, termed as LeuT-fold transporters^5,6,11,13^, inasmuch as LeuT is the first structurally solved member^15^. Structural alignment of LeuT-fold transporters unveils a similar substrate-binding site^6,11^, and a conserved cation-binding site^6,11,25,35^ known as the Na2 site in LeuT^15^. This Na2 site has been proven critical for binding or symport of the coupled substrate in LeuT-fold transporters accross various families^6,11^, listed here but not limited to, LeuT^36^ and DAT^37,38^ of the neurotransmitter:sodium symport (NSS) family, BetP^39^ and CaiT^40,41^ of the betaine/carnitine/choline (BCCT) family, vSGLT^22^ of the solute:sodium symporter (SSS) family, ApcT^25^ of the amino acid-polyamine-organocation (APC) family, and the focus of this study, Mhp1^23^ of the nucleobase:cation symporter-1 (NCS1) family (Fig. 1).

Owing to their critical functions as well as the availability of abundant structural data, LeuT-fold superfamily serves as an important system to decipher the correlation between structure, dynamics, and function of secondary active transporters. Among LeuT-fold transporters, Mhp1, the sodium-benzylhydantoin transporter from the NCS1 family, has become a popular protein for mechanistic studies of ion-coupled secondary active transporters. Mhp1 has been structurally solved in three major functional states, namely, OF Na^+^-bound state^23^, OF Na^+^/substrate-bound state^23^, and IF *apo* state^24^. With the simplest Na^+^/substrate stoichiometry (1:1)^23^, Mhp1 represents an ideal model for studying the transport and coupling mechanism in LeuT-fold transporters^24,42–47^.

At the core of the mechanism underpinning the transport function for secondary active transporters is the interplay among the driving force provided by the ions, the driven species (substrate), and the coupling provided by the conformational events of different scales during the transport cycle^5,13,35^, in other words, the mechanism by which the electrochemical potential of other ions facilitates the transport of the substrate against its concentration gradient^13^. Despite the fast growth of structural data, our understanding of the coupling mechanism in secondary active transporter is still rather preliminary. The inherently dynamic nature of the transport process, especially the coupling elements, prohibits a complete understanding of these processes solely based on the static snapshots. A mechanistic description of the transport process requires methodologies that offer treatment of the dynamics of the system. During recent years, various advanced biophysical approaches to study the dynamics of LeuT-fold transporters have yielded valuable information on the ion-coupling mechanism^36–38,44,45,47–59^. Nevertheless, a general, unifying mechanism of the coupling is still lacking.

The structural commonalities among LeuT-fold transporters, i.e., same 5-helix inverted repeats, similar substrate-binding sites, and conserved Na^+^-binding sites, strongly suggest a similar mechanism of transport^5,6,11^. However, some of the recent studies have proposed completely different ion-coupling mechanisms for different transporter families within the LeuT-fold superfamily^36,44,45,47,50,54,58–60^. A number of studies on LeuT (NSS family) using smFRET, EPR, cysteine accessibility measurements, or hydrogen/deuterium exchange mass spectrometry indicate that Na^+^ binding stabilizes the OF conformation^36,50,54,58–60^, and that such an effect requires binding of Na^+^ to the Na2 site^36,58^, a site corresponding to the only Na^+^-binding site in Mhp1. Albeit a computational study on Mhp1 (NCS1 family) proposes a similar Na^+^-binding effect as LeuT^44^, recent experimental studies using EPR, cysteine accessibility and mass spectrometry provide a conflicting picture, in which Mhp1 adopts predominantly IF conformations and Na^+^ has little effect on the conformational equilibrium of Mhp1^45,47^. The existing discrepancy in the ion-coupling mechanisms among structurally-related transporter families motivated the research presented in this article.

To understand the Na^+^-binding effects on the whole protein at the atomic level, we employed a computational paradigm that couples several state-of-the-art MD simulation approaches to study the impact of Na^+^ binding on the structure and dynamics of Mhp1 in multiple functional states and on the OF ↔ IF state transition. Microsecond-long unbiased MD simulations were performed to investigate and compare the OF *apo* to OF Na^+^-bound states, and to reveal Na^+^-binding effects on the OF state. Furthermore, we used a novel knowledge-based computational approach developed in our lab^61–64^ toward describing the OF ↔ IF conformational transition of Mhp1 and to perform an extensive exploration and identify a reliable transition pathway, in order to provide an energetic perspective on the ion-coupling mechanism.

## Results

### Na^+^ binding stabilizes the OF conformation

**Figure 2:**
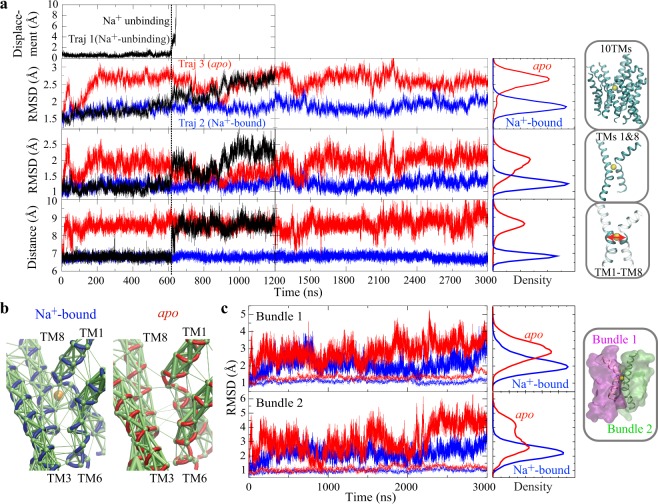
Na^+^-binding conformational effect in the OF state of Mhp1. (**a**) Dynamics and conformational changes for OF *apo* and Na^+^-bound forms. The displacement of Na^+^ ion from its binding site in Traj.1 is shown in the top panel. The second and third panels respectively depict the time series of the backbone RMSD of the 10 TMs or TMs 1 and 8 of the protein during three simulations (Traj.1: black, Traj.2: blue, Traj.3: red). The fourth panel shows the distance between TM1 and TM8 as the distance between the centers of masses of backbone atoms of residues 38 to 41 (TM1) and residues 309 to 313 (TM8). The vertical black dashed line marks the snapshot when Na^+^ unbound in Traj.1, and the vertical black solid line marks the end of Traj.1. (**b**) Dynamical network analysis for Na^+^-bound (left) and *apo* (right) states, derived from Traj.2 and Traj.3 to describe the residue-residue dynamical correlation^65^. Allosteric interactions within the network are shown as green edges weighted by correlation data. (**c**) Na^+^ locks the relative motion between the two helical bundles. Backbone RMSD of Bundle 1 (top) and Bundle 2 (bottom), either with the other bundle aligned (solid line) or with the same bundle aligned (dotted line), for *apo* (red) and Na^+^-bound (blue) forms.

In order to investigate the impact of Na^+^ binding on the conformation and dynamics of the OF state, microsecond-scale unbiased simulations were performed starting from the substrate-free, Na^+^-bound OF crystal structure of Mhp1 (PDB ID code 2JLN)^23^. During a 1.2-*μ*s unbiased simulation of Mhp1 (hereafter referred to as Traj.1), the bound Na^+^ ion spontaneously unbinds from its original binding site and diffuses into the extracellular side (unbinding observed at t = 600 ns; Fig. 2). Following Na^+^ unbinding, the core part of the Mhp1, i.e., 10 transmembrane helices (10 TMs), undergoes large-scale conformational changes. This unbinding event clearly separates the trajectory into two phases with distinct dynamical behaviors (Fig. 2), suggesting that Na^+^ binding has a strong conformational effect on the protein structure. Note that the affinity of Na^+^ for Mhp1 in the substrate-free state is not high (*K*_d_ = 1.15 ± 0.28 mM)^23^. Therefore, it is not surprising to observe spontaneous Na^+^ unbinding during the simulation.

For further comparison of the conformational and dynamical behaviors of the OF Mhp1 in the presence and absence of the bound Na^+^ ion, two additional simulations were performed, one with Na^+^ restrained in its binding site all the time (Traj.2), and the other one with the Na^+^ ion removed at the beginning of the simulation (Traj.3). Both simulations were run for 3 *μ*s.

The behaviors of these “pure” Na^+^-bound and *apo* trajectories are very consistent with the two phases observed in Traj.1, which are separated by Na^+^ unbinding (Fig. 2); in the absence of the bound Na^+^, the protein drifts away from the Na^+^-bound crystal structure and fluctuates significantly (Fig. 2), whereas in the presence of the Na^+^, Mhp1 is stable and remains close to the OF Na^+^/substrate-bound crystal structure. In the structural homolog LeuT, a recent biochemical study also suggests that occupation of Na2 stabilizes the OF conformation presumably through a direct interaction between Na^+^ and TMs 1 and 8^36^.

The two-dimensional RMSD analysis for the 10 TMs, clearly indicates that the Na^+^-bound conformational ensemble is more localized in space to the OF Na^+^/substrate-bound crystal structure (Supplementary Fig. S1). Together, these results indicate that Na^+^ binding maintains the extracellular lumen in a conformation more accessible for substrate binding from the extracellular side. This conclusion agrees with recent EPR and smFRET data of LeuT, showing that Na^+^ binding increases accessibility of the extracellular vestibule and stabilizes the OF state^48–50^.

### Na^+^ binding stabilizes substrate-binding site

**Figure 3:**
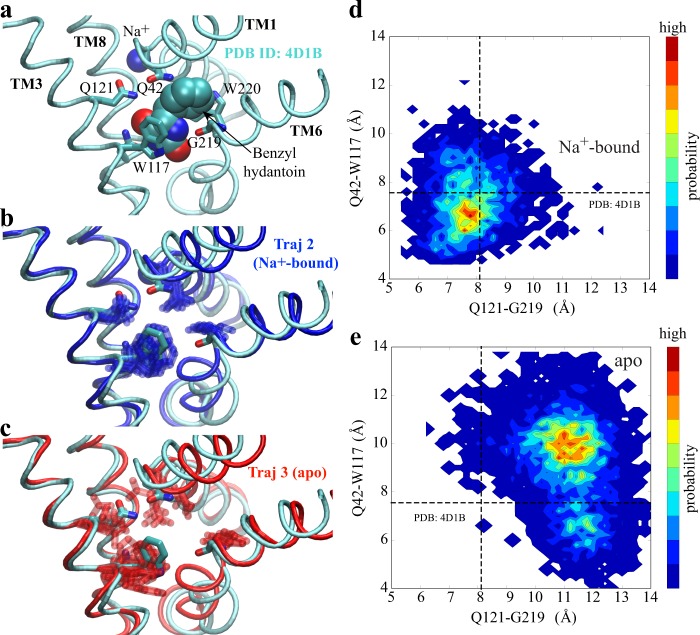
Na^+^-binding effect on the local conformation of the substrate-binding site in the OF state. (**a**) Conformation of the substrate-binding site in the OF Na^+^/substrate-bound crystal structure (PDB ID code 4D1B). The substrate is shown in VDW representation, the substrate-binding residues in stick, and the substrate-binding relevant helices in cyan tube. (**b** and **c**) Conformational dynamics of the substrate-binding site in Na^+^-bound (B, dark blue) and *apo* (C, red) trajectories. The substrate-binding residues are shown in overlapped sticks from several snapshots taken every 750 ns from respective trajectories. For clarity, only one typical conformation of the substrate-binding helices is shown in either blue (Na^+^-bound) or red (*apo*) tube, with the Na^+^/substrate-bound crystal structure shown in cyan. (**d** and **e**) Local Conformational fluctuations within Na^+^-bound (**d**) and *apo* (**e**) trajectories. As important indicators of the binding affinity, the center-of-mass distances are measured between Q121 and G219 as well as Q42 and W117. The dashed lines indicate the corresponding distances measured in the OF Na^+^/substrate-bound crystal structure (PDB ID code 4D1B).

Our simulations reveal that Na^+^ binding also stabilizes the local conformation of the substrate-binding site. It is noted that the substrate-binding site is about 10 Å from the Na^+^-binding site and there are no residues shared between the two sites. Surprisingly, in the presence of bound Na^+^, fluctuations of the substrate-binding residues are substantially reduced (Fig. 3b,c). Na^+^ binding even maintains the side-chain conformation of the residues in the substrate-binding site similar to those observed in the OF substrate-bound structure (PDB ID code 4D1B)^46^ (Fig. 3b,c).

The substrate-binding site in the OF substrate-bound structure represents a local conformation highly favorable for substrate binding. The substrate’s hydantoin moiety forms a face-to-face *π*-stacking interaction with the indole-ring of W117, and is oriented by interactions with a hydrogen bonding network formed by N318, Q121, G219, and Q42^23,46^. Previous mutagenesis studies further confirmed the critical roles of these residues in coordinating the hydantoin moiety, so that the substrate binding^46^. Thus, the conformation of the substrate-binding pocket, here measured as Q42-W117 and Q121-G219 distances, can be used as direct metrics for the affinity of the binding site. Upon Na^+^ binding, the distance distribution is much closer to that of the substrate-bound structure than the *apo* form (Fig. 3d,e). All of these data indicate that Na^+^ binding stabilizes a local conformation with increased affinity for substrate. This is consistent with the experiment that the affinity of benzylhydantoin to the protein is raised over 10-fold in the presence of saturating Na^+^ concentration^23^.

It was proposed that Na^+^ binding to the Na2 site in LeuT, corresponding to the sole Na^+^-binding site in Mhp1, plays a role in maintaining the overall OF open conformation, while Na^+^ bound at the Na1 site, which is not present in Mhp1, forms direct stabilizing interactions with the substrate^53^. However, our simulations suggest that the Na2 site, the only one Na^+^-binding site in Mhp1 and conserved in all of the Na^+^-coupled LeuT-fold symporters, not only stabilizes a global OF conformation but also keeps the substrate-binding site in local conformation favorable for substrate binding.

### Na^+^ binding to the Na2 site restrains the relative motion between the two helical bundles

Detailed analysis based on our simulations shed light on the underlying molecular mechanism of how Na^+^ binding also stabilizes the global conformation of the protein. The Na^+^ ion bound in the Na^+^-binding site, coordinated with residues from TM1 and TM8, could restrain the distance between these two helices and thereby constrain the relative orientation and movement of the two helical bundles (Fig. 2).

Dynamical network analysis^65^ was also performed on Traj.2 and Traj.3 to describe the residue-residue dynamical correlation, and to characterize the communication network within the protein. In the presence of the bound Na^+^ ion, there is a stronger allosteric coupling among TMs 1, 3, 6, and 8 (Fig. 2b), including hydrophobic interactions and hydrogen bonds. These interactions hold the four substrate-binding helices, TMs 1, 3, 6, and 8, together as a stable core of the protein. In the absence of Na^+^, the extent of coupling among these helices is significantly reduced, especially between TM3 and TM6 (Fig. 2b). The residues forming the substrate-binding site are all from these four TMs (Fig. 3a), therefore their stabilization keeps the substrate-binding site in a stable conformation with high binding affinity. Considering that these four TMs are conserved structural elements, the long-range (~10 Å) coupling between Na^+^ and substrate-binding sites is expected to be a common feature among LeuT-fould transporters. This notion is in line with recent experimental reports in LeuT and in human Na^+^ coupled glucose transporter (hSGLT1) where Na^+^ binding to the Na2 site is shown to increase substrate binding affinity^36,66^.

It has been proposed that the alternating-access model of Mhp1 might be described as a rigid-body rotation of the two helical bundles, TMs 3, 4, 8, and 9 (Bundle 1) relative to TMs 1, 2, 6, and 7 (Bundle 2)^24^. It is noted that TMs 1, 3, 6, 8, where Na^+^-binding residues are located, belong to the two bundles. Located at the interface of the two helical bundles, thereby the Na2 site with a bound Na^+^ holds these four TMs together, stabilizing the two helical bundles, constraining the global conformation of the protein in an OF conformation. It is confirmed by RMSD analysis that the helical bundles move much less relative to the each other upon Na^+^ binding (Fig. 2c). Therefore, Na^+^ binding in Mhp1 results in two synergistic effects on the transporter, namely, stabilizing an OF open conformation, and tightening the substrate-binding site to achieve a higher binding affinity.

### Na^+^ binding increases the energy barrier along OF ↔ IF transition

In addition to the effects on the OF state, Na^+^ binding might also impact the energetics associated with the OF ↔ IF transition in Na^+^-coupled symporters. As the species providing the driving force for transport, Na^+^ is expected to affect the free energy associated with the transition. Here, we have used a knowledge-based computational approach^61–63^ toward describing the OF ↔ IF conformational transition of Mhp1, using driven simulations employing system-specific collective variables combined with nonequilibrium work measurements to provide a qualitative assessment. This approach has been employed to characterize OF ↔ IF conformational changes for several transporters^61–63^.

**Figure 4:**
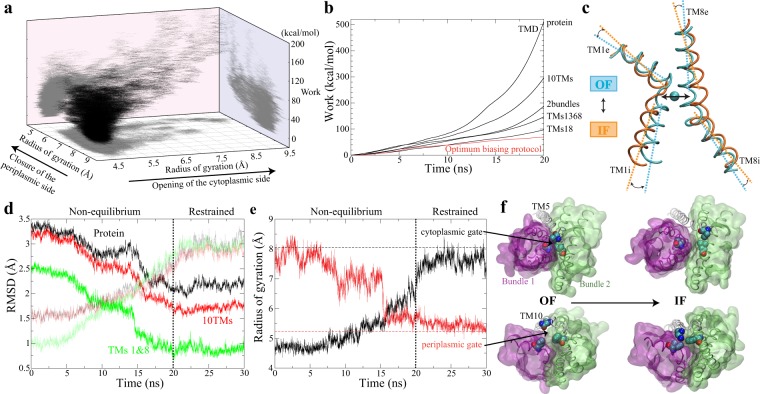
Identification of the optimal protocol to induce the OF ↔ IF global transition. (**a**) 70 independent biased simulations were performed to explore the transition pathway. Trajectories are shown in the space formed by the radius of gyration of both periplasmic and cytoplamsic gates, and the associated nonequilibrium work. (**b**) Representative examples of biasing protocols to demonstrate how nonequilibrium work was reduced by optimizing the collective variables. The black lines represent the nonequilibrium work associated with simulations using RMSD as collective variables for different structural elements. The red line shows the nonequilibrium work of simulation using the optimal protocol with collective variables shown in (**c**). (**c**) The collective variables used in the optimal protocol: the distance between TM1 and TM8, the angle between TM1e and TM8e, and the angle between TM1i and TM8i. The differences of these collective variables between OF and IF states are shown in the comparison of two Na^+^-binding helices, TMs 1 and 8, between OF Na^+^-bound (PDB ID code 2JLN) and IF *apo* (PDB ID code 2X79) crystal structures. (**d**) The backbone RMSDs of different structural elements during the biasing simulation with the optimal protocol and the following restrained simulation, respectively with reference to OF Na^+^-bound (PDB ID code 2JLN) (dark lines) and IF *apo* (PDB ID code 2X79) (semi-transparent lines) crystal structures. (**e**) The radius of gyrations of cytoplasmic (black) and periplasmic (red) gates in the same trajectory shown in (**d**). The radius of gyration of the two gates in IF *apo* crystal structure (PDB ID code 2X79) are shown as horizontal dashed lines. The cytoplasmic gating residues include I161, I230, and L320, and the periplasmic gating residues include I47, F120, and N360. (**f**) The opening of the cytoplasmic gate (top), and the closure of the periplasmic gate (bottom) during the OF to IF transition. The gating residues are shown in VDW, and the two bundles are shown in transparent surfaces. TM5 and TM10 are shown as white tubes.

Based on structural data available for Mhp1^23,24^, we designed a variety of distinct reaction coordinates, i.e., orientations, RMSDs, radius of gyrations and distances of different structural elements (described in Methods and summerized in Table 1), to trigger a relative movement of the two bundles and to achieve the global OF ↔ IF transition. In order to optimize the biasing protocol, ~70 biased simulations (totalling to more than 2 *μ*s) were performed (Fig. 4a). The optimized biasing protocol was identified based on three criteria: (i) complete OF ↔ IF transition, (ii) minimal nonequilibrium work (Fig. 4b), and (iii) mechanistic relevance. TMs 1 and 8 are the smallest structural elements identified to be able to trigger a complete OF ↔ IF transition. Driving TMs 1 and 8 from OF to IF configuration, using either (i) RMSD or (ii) a combination of their orientation and interhelical distance (Fig. 4c) as collective variables, induces the global OF ↔ IF transition in repeated independent simulations (Fig. 4d–f and Table 1). Among all of the biasing protocols that induced complete OF ↔ IF transition, one that consistently required least work is the orientation of TMs 1 and 8 (*θ*) combined with their distance (Figs. 4b and 1), not only for the Na^+^-bound but also for the *apo* form. As noted, TMs 1 and 8 respectively belong to Bundles 1 and 2, and are also the helices that form the binding site for the Na^+^ ion (Fig. 4c), which provides the driving force for the transport. This observation is consistent with our results on both Mhp1 and its homolog in that the local conformational change of TMs 1 and 8 is highly correlated with the global OF ↔ IF transition^67^. Therefore, it is not surprising that this biasing protocol also shows high mechanistic relevance to the OF ↔ IF transition.

**Table 1:** List of the biased driven simulations performed for the OF ↔ IF transition, induced using different biasing protocols.

State	Protocol	Structural elements	Number of copies*	Runtime (ns)^†^	Transition^‡^	Total work (kcal/mol)
Na^+^-bound	RMSD	protein^¶^	1	20	yes	511
Na^+^-bound	RMSD	TMs 1-10	1	20	yes	296
Na^+^-bound	RMSD	TMs 1-4,6-9	1	20	yes	188
Na^+^-bound	RMSD	TMs 1,3,6,8	1	20	yes	147
Na^+^-bound	RMSD	TMs 3,6	1	20	no	—
Na^+^-bound	RMSD	TMs 1,3	1	20	no	—
Na^+^-bound	RMSD	TMs 6,8	1	20	yes	106
Na^+^-bound	RMSD	TMs 1,8	3	5	yes	141 ± 18
Na^+^-bound	RMSD	TMs 1,8	2	20	yes	98 ± 0.45
*apo*	RMSD	TMs 1,8	3	5	yes	121 ± 6
*apo*	RMSD	TMs 1,8	2	20	yes	100 ± 5
Na^+^-bound	*θ*^§^	TMs 1,8^#^	1	20	no	—
Na^+^-bound	*d*^§^	Na^+^ site^||^	2	20	no	—
Na^+^-bound	RMSD + *θ*	Na^+^ site + TMs 1,8	6	20	yes	78 ± 8
*apo*	RMSD + *θ*	Na^+^ site + TMs 1,8	6	20	yes	73 ± 4
Na^+^-bound	*R*_*g*_ + *θ*^§^	Na^+^ site + TMs 1,8**	9	20	partial	—
*apo*	*R*_*g*_ + *θ*	Na^+^ site + TMs 1,8	2	20	partial	—
Na^+^-bound	*d* + *θ*	Na^+^ site + TMs 1,8	6	5	yes	93 ± 16
Na^+^-bound	*d* + *θ*	Na^+^ site + TMs 1,8	9	20	yes	78 ± 7
*apo*	*d* + *θ*	Na^+^ site + TMs 1,8	2	5	yes	91 ± 0.01
*apo*	*d* + *θ*	Na^+^ site + TMs 1,8	12	20	yes	69 ± 6

The work profiles associated with representative simulations are shown in Fig. 4.

^*^Different force constants were tested and tuned when multiple copies were performed for the same collective variable.

^†^Here runtime is just for the driven portion of the simulation, which is followed with restrained equilibrium and nonrestrained simulations for relaxation at the target state in most cases. The restrained equilibrium and nonrestrained simulations are always the same length or longer than the driven simulation runtime.

^‡^The radius of gyrations of cytoplasmic and periplasmic gates were measured to evaluate the completion of the conformational transition of the transporter from OF to IF state. “partial” means that transition was achieved in a fraction of multiple copies.

^§^*θ*: relative orientation; *d*: distance; *R*_*g*_: radius of gyration.

^¶^C_*α*_ atoms are selected for specific structural elements when RMSD is the collective variable.

^#^Backbone heavy atoms are selected for specific structural elements when *θ* is the collective variable.

^||^The distance of Na^+^-binding site is represented by two distances respectively between C_*α*_ atoms of A38 and A309, and those of I41 and T313.

**The radius of gyration of Na^+^-binding site is defined by C_*α*_ atoms of residues forming the site, i.e., A38, I41, A309, S312, and T313.

**Figure 5:**
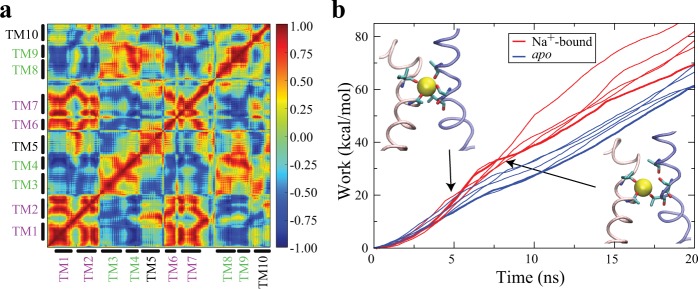
Na^+^-binding impact on the OF ↔ IF transition. (**a**) The dynamic correlation analysis based on the biased simulation with the optimal protocol reveals the high correlation between the two helical bundles: Bundle1 (TMs 1, 2, 6, and 7) and Bundle2 (TMs 3, 4, 8, and 9). (**b**) Using the same biasing (optimal) protocol, the non-equilibrium work for the OF ↔ IF transition is reproducibly lower in *apo* than the Na^+^-bound form. The molecular images are representative snapshots selected before (upper left) and after (lower right) the breaking of the Na^+^-binding site, with the bound Na^+^ shown in VDW, the substrate-binding residues in stick, and TM1 and TM8 in pink and blue tubes respectively.

The OF ↔ IF transition in all of these simulations, as shown in the Fig. 5a, can be described as a relative rigid-body rotation of the two helical bundles. During the large-scale conformational change of OF ↔ IF transition, the movements of the helices within each bundle are highly correlated (Fig. 5a). This is also consistent with the previously hypothesised alternating-access model that the two helical bundles are approximately rigid bodies^24^. All of these data suggest that the biasing protocol based on the orientation and distance of TMs 1 and 8 captures major structural elements involved in the transition, and therefore provides a reliable pathway to characterize the OF ↔ IF transition.

The same optimal biasing protocol was used to induce the OF ↔ IF transition in both *apo* and Na^+^-bound forms. To compare the pattern and trend of work, rather than individual simulation profile, we repeated the simulations in multiple copies in the presence and absence of Na^+^ in the binding site. In these simulations, the non-equilibrium work for OF ↔ IF transition is reproducibly lower in *apo* than the Na^+^-bound form (Fig. 5b). More importantly, based on the trend of work, there is a consistent hump (between 5 and 7 ns) in non-equilibrium work profiles in the Na^+^-bound form, which results in more cumulative work than the *apo* form (Fig. 5b). This hump is highly correlated with the breaking of the Na^+^-binding site in the presence of bound Na^+^ ion (Fig. 5b). The biased simulations using the optimal biasing protocol allow us to evaluate how the free energy barrier along the OF ↔ IF transition is affected by Na^+^ binding. It suggests that Na^+^ binding raises the energy barrier for the OF ↔ IF transition. Lowering the transition rate upon Na^+^ binding is in agreement with the previous smFRET measurements in homologous LeuT where increasing Na^+^ concentration resulted in a decrease of the frequency of OF ↔ IF transition^50^.

## Discussion

The Na2 site is a highly conserved structural element in both Na^+^-dependent and -independent LeuT-fold transporters. It is the only conserved Na^+^-binding site in all Na^+^-dependent LeuT-fold transporters, namely, LeuT^15^, vSGLT^21^, Mhp1^23^, BetP^68,69^, DAT^28^, MhsT^29^, and SERT^31^, albeit multiple Na^+^-binding sites might exist in some members. More interestingly, the solved structures and biochemical characterization in several Na^+^-independent transporters have also revealed a basic residue located in a position equivalent to the Na2 site, namely, a lysine residue (K158) in the proton-coupled amino acid transporter ApcT^25^, and an arginine residue (R262) in carnitine/gamma-butyrobetaine antiporter (CaiT)^40,41^. These residues serve a similar functional role as the Na^+^ in Na^+^-dependent transporters^25,41^. Located at the same position as the interface between the two helical bundles (Fig. 1), the conserved Na2 site among LeuT-fold transporters, regardless of Na^+^-dependence or direction of transport (symporters or antiporters), strongly implies a conserved functional role of this site in the transport mechanism.

A long-lived puzzle in such a delicate secondary active transport machinery is the coupling between the two transported species, namely, how the energy stored in the Na^+^ electrochemical gradient drives the uphill transport of the substrate. Our MD simulations on Mhp1 reported in this study unveil that, rather than simply passing a kinetic impulse to the transporter, Na^+^ binding to the Na2 site exhibits multiple effects which synergistically facilitate the substrate transport. The results demonstrate how Na^+^ binding in the Na2 site restrains the two helical bundles in the OF state, which brings at least two Na^+^-binding effects on the transporter to couple the downhill Na^+^ permeation to the uphill substrate transport. The first effect is an allosteric coupling in that Na^+^ binding stabilizes substrate-binding residues in an OF conformation favorable for substrate binding. This substantiates the biochemical assays on Mhp1^23^, LeuT^36^, and hSGLT1^66^ showing that Na^+^ binding in the Na2 site substantially increases the substrate-binding affinity. The higher affinity upon Na^+^ binding enables the transporter to bind the substrate from a lower-concentration environment.

**Figure 6:**
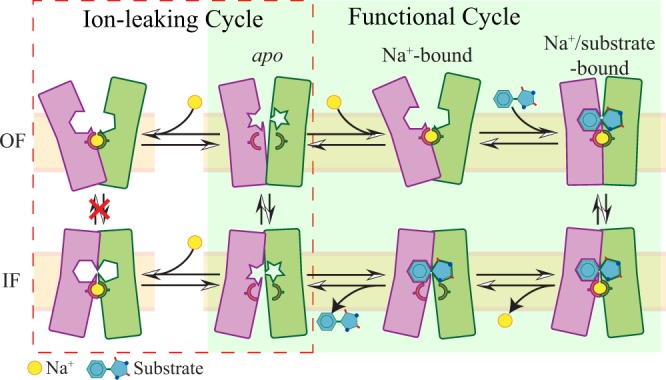
Schematics of Na^+^-binding effects on LeuT-fold transporters. In a functional transport cycle (light green region), the transporter (two helical bundles shown in purple and green rectangles) traverses OF *apo* (2nd column, top), OF Na^+^-bound (3rd column, top), OF Na^+^/substrate-bound (4th column, top), IF Na^+^/substrate-bound (4th column, bottom), IF substrate-bound (3rd column, bottom), and IF *apo* (2nd column, bottom) states, schematically indicated by the relative angle between two helical bundles and the loading state of Na^+^ (yellow sphere) and substrate. Compared to the OF *apo* state, Na^+^ binding stabilizes the substrate-binding site (the excavated shapes in the helical bundles) in a local conformation favorable for substrate binding (upper middle). Meanwhile, Na^+^ binding to the OF *apo* and IF *apo* states introduces an extended ion-leaking cycle (red line circled region), in which the OF ↔ IF transition is disfavored to avoid energy dissipation (1st column). Such OF ↔ IF transition is allowed in the *apo* form (2nd column) and in the Na^+^/substrate-bound form (4th column) in the functional transport cycle.

The second effect, observed in both unbiased and biased simulations, is the influence of Na^+^ binding on the kinetics. Highly consistent with the rocking-bundle mechanism^34^, Na^+^ binding at the Na2 site is found to restrain the motion of two helical bundles, increasing the energy barrier of the OF ↔ IF transition (Fig. 6). This computational observation is also in agreement with the smFRET studies on LeuT showing that increasing Na^+^ concentration could decrease the frequency of OF ↔ IF transitions^50,59^. This kinetic effect keeps the transporter from undergoing OF ↔ IF transition with only the bound Na^+^ (Fig. 6), thereby, preventing the leak of Na^+^ ions.

Another potential effect is a thermodynamic impact, in which Na^+^ binding might change the free energy difference between OF and IF states, and shift the conformational equilibrium toward the OF state. Whether this thermodynamic impact is a general effect within the LeuT-fold superfamily is still controversial. The studies on LeuT consistently suggest Na^+^ binding stabilizes the OF conformation^36,50,54,58–60^, whereas experimental measurements on Mhp1 show that Na^+^ has little effect on the conformational equilibrium^45,47^. Different from previous experimental studies on Mhp1, our computational results indicate that Mhp1 might still benefit from a similar Na^+^-binding effect as LeuT by stabilization of the OF conformation. It should be noted that both EPR^45^ and mass spectrometry combined with cysteine accessibility^47^ indicate that Mhp1 predominantly adopts an IF conformation in both Na^+^-free and Na^+^-rich environments. Major peaks in distance distributions of EPR spin labels also match with the corresponding distance distributions from our simulations in the IF *apo* state (Supplementary Fig. S2). Considering previous studies suggesting that Na^+^ binding is less favorable in the IF state for several LeuT-fold transporters^19,24,27,38,67^, and the fact that Na^+^ is absent in the Na2 site in all available IF open structures of LeuT-fold transporters^19,22,24,27^, it is reasonable that the major peak in distance distribution of the IF state is not substantially affected by Na^+^ binding to the Na2 site. Meanwhile, the possibility that Na^+^ binding still stabilizes the OF conformation cannot be excluded from these data, as the signal of Na^+^-binding effect in the OF state might be masked by the predominant population of the IF state. Probably the contribution made by Na^+^ binding to the OF state is still much less than the free energy difference between IF and OF states, thus Na^+^ binding cannot significantly shift Mhp1 conformational equilibrium. Above all, to clarify this discrepancy between LeuT and Mhp1 reports^36,45,47,50,54,58,59^, characterization of the free energy profiles along the OF ↔ IF transition pathway in the presence/absence of Na^+^ would be required for multiple LeuT-fold transporters.

Conceptually, the Na2 site serves as an asymmetric element in the symmetric structural topology, contributing to the directional transport. The function of secondary active transporters requires a special structural basis for their transport against substrate chemical gradient through the membrane under physiological conditions. Most secondary active transporters, including the LeuT-fold superfamily, exhibit a pseudo-two-fold symmetry between the two halves of the protein. In principle, there should be some asymmetric features to differentiate the two major conformational states. In the Na^+^-dependent LeuT-fold transporters, the conversed Na2 site likely plays the role of a general asymmetrical element in the symmetric topology. The Na2 site, located between TMs 1 and 8, specifically binds Na^+^ in the OF conformation (Fig. 1b), favoring substrate binding to this state. However, besides BetP^68^, there is no such equivalent Na^+^-binding site located in a symmetric position between TM3 and TM6, to provide similar effects in the IF state. Structural-based sequence alignment with the LeuT-fold symporters supports the vital role of the Na2 site (Supplementary Fig. S3). There is a typical “(S/T)-(S/T)” motif in TM8, a conserved feature of the Na2 site, in all of the Na^+^-coupled or H^+^-coupled LeuT-fold transporters, whereas an equivalent and conserved motif is absent in TM3. Therefore, the Na2 site, the unique conserved Na^+^-binding site only located between TM1 and TM8, is one of the structural determinants for the function of the LeuT-fold transporters.

In summary, our results reveal synergistic Na^+^-binding effects on Mhp1 at the atomic level. Na^+^ binding in the Na2 site restrains the two helical bundles in the OF state, stabilizes an OF conformation favorable for substrate binding, and also increases the energy barrier against the OF ↔ IF transition. The combination of these allosteric, kinetic, and thermodynamic Na^+^-binding effects altogether increases the lifetime of the OF conformation with a high affinity for substrate binding, ultimately, facilitating recruitment of the substrate from a low-concentration environment to fulfill the active transport function. Considering that both the Na2 site and the two helical bundles are highly conserved in the LeuT-fold transporters, we propose that these common Na^+^-binding effects may contribute to a conserved ion-coupling mechanism in this superfamily. Furthermore, the observation that the bound Na^+^ facilitates substrate recruitment in Mhp1 makes one wonder whether a more general mechanism in which the binding of the energy-providing species is directly coupled to the recruitment of the driven substrate might exist among ion-coupled transporters.

The observed triple Na^+^-binding effects, altogether, suggest that the Na^+^ gradient reshapes the free energy landscape to promote an inward substrate transport. In such an indirect manner, the energy stored in the Na^+^ gradient is transferred to potential energy—rather than kinetic energy—then transformed into the driving force for the substrate.

## Methods

### Model building

The simulation systems were constructed by embedding Mhp1, which was taken from either substrate-free, Na^+^-bound OF crystal structure (PDB ID code 2JLN)^23^, or *apo* IF crystal structure (PDB ID code 2X79)^24^, into a lipid bilayer, as described in detail below. The titration states of ionizable residues (aspartate, glutamate, lysine, arginine, histidine, and tyrosine) were assigned based on pK_*a*_ calculations performed using the H++ server^70^, which resulted in a model in which all residues have their default titration states. The first principal axis of the protein was aligned with the *z* axis using the OPM (Orientations of Proteins in Membranes) database^71^. Then, the system was inserted into a patch of POPE (1-palmitoyl-2-oleoyl-*sn*-glycero-3-phosphatidylethanolamine) bilayer (100 × 100 Å^2^) generated using the Membrane Builder plugin of VMD^72^ with the membrane normal along the *z*-axis. The lipid molecules overlapping with the protein were deleted. The system was solvated using the program SOLVATE^73^, and water molecules in the lipid-protein interface were deleted. The simulation system was then neutralized with 100 mM NaCl using the Autoionize plugins of VMD^72^. The final dimensions of the system before equilibration were 98 × 98 × 100 Å^3^ including ~82,000 atoms.

### Conventional simulation protocol

All the unbiased simulations were performed using NAMD 2.6^74^ or Desmond on Anton^75^. The CHARMM27 force field^76^, including the CMAP correction^77^, was used for proteins, whereas lipids were described with the CHARMM36 force field^78^. Explicit water was described with the TIP3P model^79^. All the simulations were performed under periodic boundary conditions with a time step of 2 fs. Throughout the simulations, bond distances involving hydrogen atoms were fixed using the SHAKE algorithm^80^.

After initial minimization of at least 1000 steps, all systems were simulated using the following protocol: (1) 0.5 ns NVT simulation with all atoms fixed except for the acyl chains of the lipid molecules, in order to introduce a higher degree of disorder in the lipid tails; (2) simulation in an NPT ensemble with positional restraints (k = 2 kcal/mol/Å^2^) applied to all protein and substrate atoms; and (3) equilibration in an NPT ensemble, without restraints. After the initial equilibration, the systems were subjected to production simulations in the NPT ensemble. For MD simulations using NAMD, constant temperature was maintained by employing Langevin dynamics with a damping coefficient of 0.5 ps^−1^. The Langevin piston method^81,82^ was employed to maintain a constant pressure of 1.0 atm with a piston period of 100 fs. Short-range non-bonded interactions were calculated using a cutoff distance of 12 Å, and long-range electrostatic interactions were calculated using the particle mesh Ewald (PME) method^83^. For MD simulations using Desmond on Anton^75^, the Berendsen coupling scheme was employed to maintain a constant pressure of 1.0 atm, and long-range electrostatic interactions were computed using the *k*-space Gaussian split Ewald method^84^, with a 64 × 64 × 64 grid.

Four microsecond-scale unbiased simulations were performed on Anton, in which three were in the OF state, and the fourth was in the IF state. The first one (Traj.1) was started from the substrate-free, Na^+^-bound OF crystal structure of Mhp1 (PDB ID code 2JLN)^23^ without any restraints for Na^+^ during the production simulation (1.2 *μ*s). The second one restrained the Na^+^ ion in its binding site throughout the 3 *μ*s of the simulation (Traj.2). The Na^+^ ion was restrained using the “ExtraBonds” feature in NAMD, by applying in-total five harmonic distance restraints (k = 200 kcal/mol/Å^2^) between the Na^+^ ion and the carbonyl oxygen atoms of A38, I41, and A309, as well as the hydroxyl oxygen atoms of the side chains of S312 and T313, with reference to those distances measured in Na^+^-bound OF crystal structure (PDB ID code 2JLN). In the third simulation, the Na^+^ ion was removed at the beginning (Traj.3, 3 *μ*s). The fourth trajectory (Traj.4) was started from the *apo* IF crystal structure of Mhp1 (PDB ID code 2X79)^24^ and ran for 3 *μ*s.

### Biased simulation to characterize OF ↔ IF Transition

A sampling strategy using biased MD simulations along mechanistically relevant, system-specific reaction coordinates developed in out lab was applied to characterize large-scale structural transitions of several membrane transporters^61–64^. The approach provides an empirical framework for optimizing the biasing protocols in a series of short all-atom simulations. By using advanced system-specific biasing protocols, it has been shown that the effectiveness in sampling complex transition pathways and searching the optimal pathway could be improved significantly^61–63^.

This approach to explore the optimal reaction coordinate (biasing protocol) is based on an initial extensive empirical search. Our previous studies^43,67,85,86^ on Mhp1 and its homolog allowed us to limit the conformational sampling to a subspace highly relevant to structural transition. We designed a wide range of mechanistically relevant, system-specific reaction coordinates, i.e., orientations, RMSDs, radius of gyrations (*R*_*g*_) and distances (*d*) of different structural element (listed in Table 1), to do an extensive exploration of the space and optimization of the biasing protocol. Their usefulness and applicability to induce the transition of interest are examined by conformational analysis using IF crystal structure (PDB ID code 2X79)^24^ as a reference, and then qualitatively assessed by nonequilibrium work measurements^61,62^.

~70 biased simulations were performed based on distinct biasing protocols (listed in Table 1). In most cases, biased simulations were followed by restrained MD simulations in which the system is subject to a time-independent biasing potential centered at the final target, and then were further equilibrated without any bias to verify the completion of the transition. These simulations total to more than 2 *μ*s. The optimized biasing protocol involves the collective variables, i.e., four orientation quaternions describing the orientation of two unwound transmembrane helices TMs 1 and 8, and the distance between them (Fig. 4c).

This optimized biasing protocol consistently requires least nonequilibrium work among all the biasing protocols, not only for the Na^+^-bound but also for the *apo* state. Thus the same biasing protocol was applied for *apo* and Na^+^-bound forms when baising simulations were repeated during the production phase (Table 1), to achieve a reliable assessment and comparison between the OF ↔ IF transitions in the presence and absence of Na^+^ ion in its binding site^87^.

## Supplementary information

Supplementary Information
