# Supplementary material for: Locking Two Rigid-body Bundles in an Outward-Facing Conformation: The Ion-coupling Mechanism in a LeuT-fold Transporter
Source: Sci Rep. 2019 Dec 20;9:19479. doi: 10.1038/s41598-019-55722-6 (PMC6925253; doi:10.1038/s41598-019-55722-6)
Supplement: Supplementary file 1 — Supplementary Information [file 41598_2019_55722_MOESM1_ESM.pdf]

Supplementary Information for: Locking Two Rigid-body Bundles in an Outward-Facing Conformation: The Ion-coupling Mechanism in a LeuT-fold Transporter

Jing Li[1,2,3,4,+], **Zhiyu Zhao**[1,2,3,4], **and Emad Tajkhorshid**[1,2,3,4,*]

[1]NIH Center for Macromolecular Modeling and Bioinformatics, University of Illinois at Urbana-Champaign, Urbana, IL 61801
[2]Beckman Institute for Advanced Science and Technology, University of Illinois at Urbana-Champaign, Urbana, IL 61801
[3]Department of Biochemistry, University of Illinois at Urbana-Champaign, Urbana, IL 61801
[4]Center for Biophysics and Quantitative Biology, University of Illinois at Urbana-Champaign, Urbana, IL 61801
[+]Present address: Department of Biochemistry and Molecular Biology, University of Chicago, Chicago, IL 60637
[*]Correspondence and requests for materials should be addressed to E.T. (email: emad@life.illinois.edu)

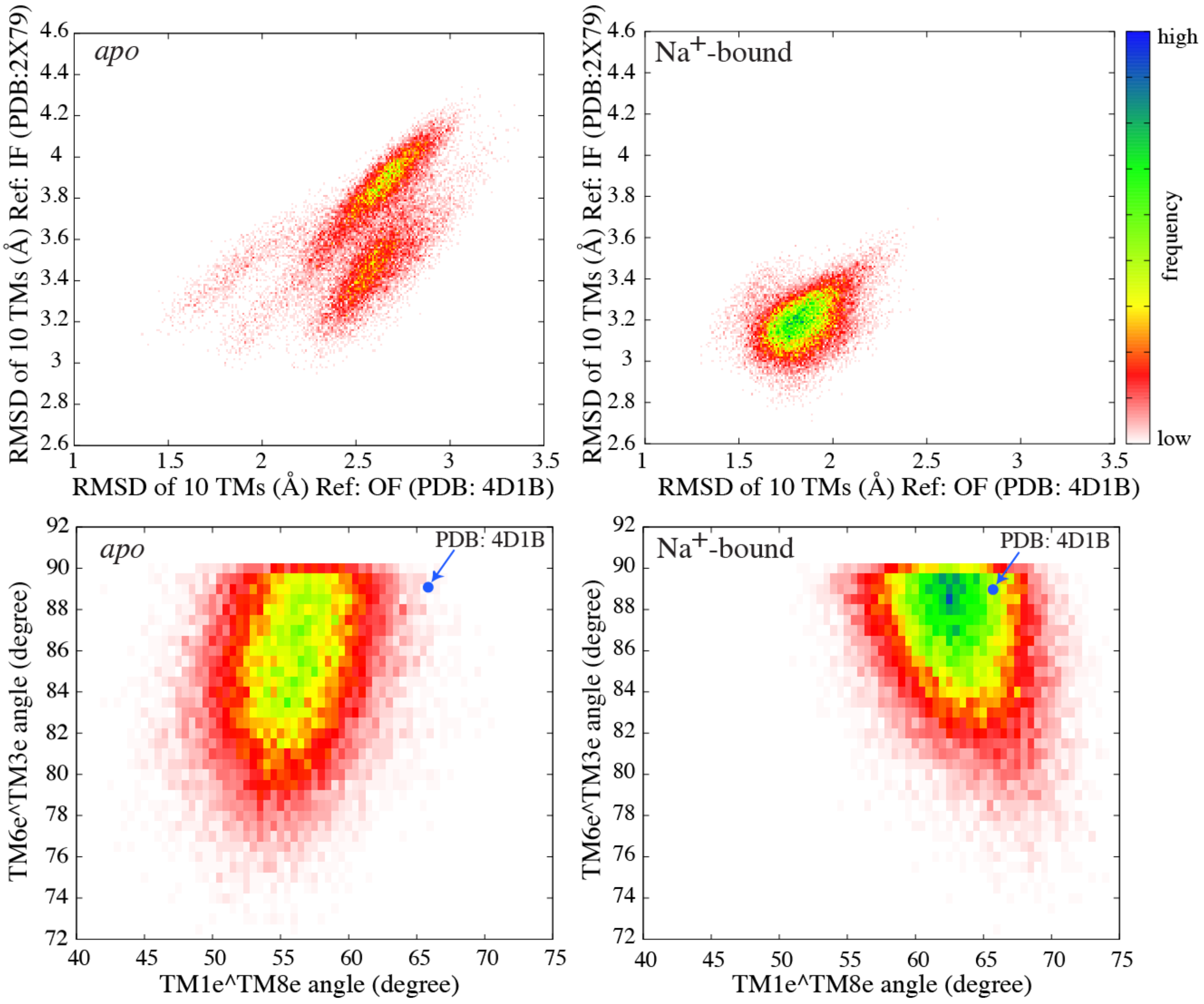

Figure S1. Na^+-binding effect on the global protein conformation and dynamics in the OF state. (Top) Distribution of backbone RMSDs of 10 TMs in the *apo* (Traj.3, left) and Na^+-bound (Traj.2, right) simulations with reference to OF Na^+/substrate-bound (PDB ID code 4D1B) and IF *apo* (PDB ID code 2X79) crsytal structures. (Bottom) Distribution of angles between TM1e and TM8e (x axis) and TM3e and TM6e (y axis) in the *apo* (Traj.3, left) and Na^+-bound (Traj.2, right) trajectories.

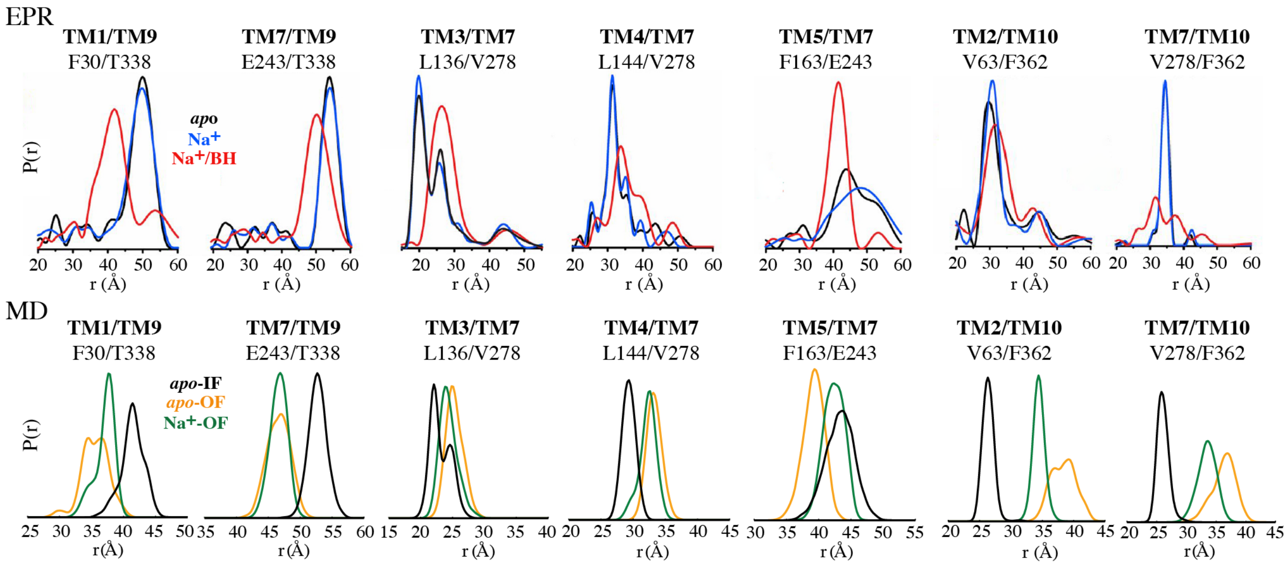

Figure S2. Comparison of EPR distance distributions adapted from Kazmier et al.[1] with permission (copyright 2014 National Academy of Sciences) (top), and the distance distributions calculated from MD simulations of IF apo, OF apo, and OF Na^+-bound states (Traj.4, Traj.3, and Traj.2 respectively) (bottom). EPR distance distributions for each pair were obtained in the *apo*, Na^+-bound (Na^+), and Na^+- and benzyl-hydantoin-bound (Na+/BH) intermediate states. The distances from MD simulations are those between the last heavy atoms on the side chain of the two residues (CZ for Phe, CG2 for Thr and Val, OE2 for Glu, CD2 for Leu), which are expected to be slightly deviated from those between the EPR spin labels in experiments. Both distributions from EPR experiments and MD simulations are shown as a probability, P(r), relative to distance, r.

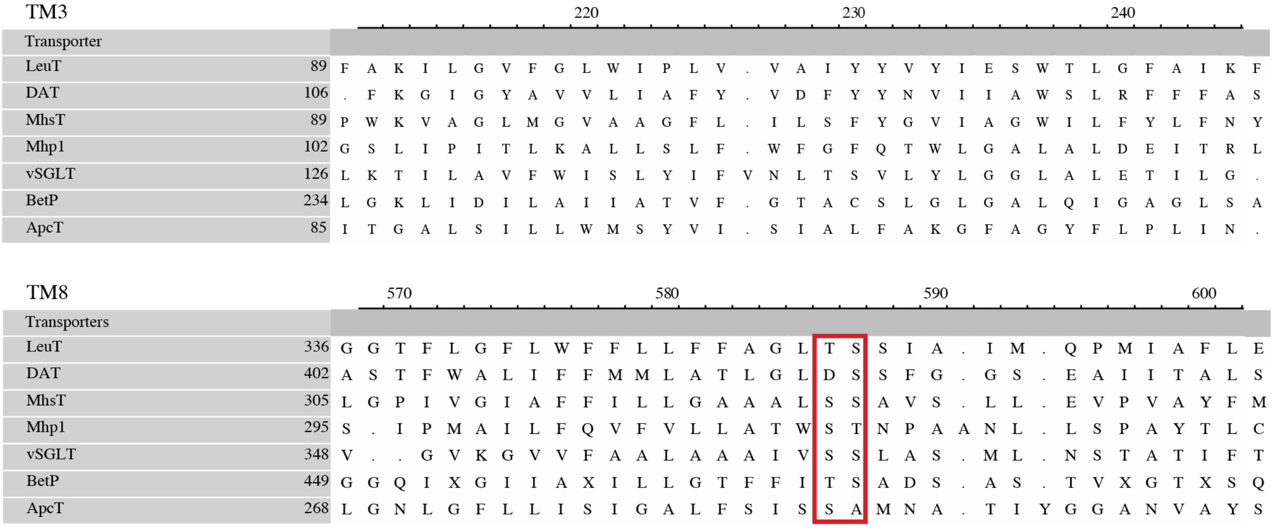

Figure S3. Structural based sequence alignment for the symporters in LeuT-fold transporters, for the TM3 (top panel) and the TM8 (bottom panel). The conserved motif in TM8 for the Na2 site is highlighted in the red box. The sequence alignment is performed with the MultiSeq module of VMD and using Stamp Structural Alignment.

References

1. Kazmier, K., Sharma, S., Islam, S. M., Roux, B. & Mchaourab, H. S. Conformational cycle and ion-coupling mechanism of the Na^+/hydantoin transporter Mhp1. *Proc. Natl. Acad. Sci. USA* **111**, 14752–14757 (2014).
